# Progression-free and overall survival in ovarian cancer patients treated with CVac, a mucin 1 dendritic cell therapy in a randomized phase 2 trial

**DOI:** 10.1186/s40425-016-0137-x

**Published:** 2016-06-21

**Authors:** H. J. Gray, B. Benigno, J. Berek, J. Chang, J. Mason, L. Mileshkin, P. Mitchell, M. Moradi, F. O. Recio, C. M. Michener, A. Alvarez Secord, N E. Tchabo, J. K. Chan, J. Young, H. Kohrt, S. E. Gargosky, J. C. Goh

**Affiliations:** University of Washington Medical Center, Seattle, WA USA; Northside Hospital, Atlanta, GA USA; Stanford Women’s Cancer Center, Stanford, CA USA; Marin Cancer Care, Greenbrae, CA USA; Scripps Cancer Center, San Diego, CA USA; Peter MacCallum Cancer Centre, East Melbourne, Vic Australia; Olivia Newton-John Cancer and Wellness Centre, Austin Health, Heidelberg, Vic Australia; New York Downtown Hospital, New York, NY USA; South Florida Center for Gynecologic Oncology, Boca Raton, FL USA; Cleveland Clinic Foundation, Cleveland, OH USA; Duke Cancer Institute, Duke University Health System, Durham, NC USA; Morristown Medical Center, Morristown, NJ USA; University of California, San Francisco & Sutter Health Research Institute, San Francisco, CA USA; Medical University of South Carolina, Charleston, SC USA; Stanford University Cancer Institute, Stanford, CA USA; Prima BioMed, Sydney, NSW Australia; Greenslopes Private Hospital, Royal Brisbane & Women’s Hospital, University of Queensland & Gallipoli Research Foundation, Greenslopes, QLD Australia

**Keywords:** Dendritic cells, Mucin 1, Ovarian cancer, Maintenance, Immunotherapy

## Abstract

**Background:**

CAN-003 was a randomized, open-label, Phase 2 trial evaluating the safety, efficacy and immune outcomes of CVac, a mucin 1 targeted-dendritic cell (DC) treatment as a maintenance therapy to patients with epithelial ovarian cancer (EOC).

**Methods:**

Patients (*n* = 56) in first (CR1) or second clinical remission (CR2) were randomized (1:1) to standard of care (SOC) observation or CVac maintenance treatment. Ten doses were administered over 56 weeks. Both groups were followed for progression-free survival (PFS) and overall survival (OS).

**Results:**

Fifty-six patients were randomized: 27 to SOC and 29 to CVac. Therapy was safe with only seven patients with Grade 3–4 treatment-emergent adverse events. A variable but measurable mucin 1 T cell-specific response was induced in all CVac-treated and some standard of care (SOC) patients. Progression free survival (PFS) was not significantly longer in the treated group compared to SOC group (13 vs. 9 months, *p* = 0.36, hazard ratio [HR] = 0.73). Analysis by remission status showed in the CR1 subgroup a median PFS of 18 months (SOC) vs. 13 months (CVac); *p* = 0.69 (HR = 1.18; CI 0.52–2.71). However CR2 patients showed a longer median PFS in the CVac-treated group (median PFS not yet reached, >13 vs. 5 months; *p* = 0.04, HR = 0.32 CI). OS for CR2 patients at 42 months of follow-up showed a difference of 26 months for SOC vs. > 42 months for CVac-treated (as median OS had not been reached; HR = 0.17 (CI 0.02–1.4) with a *p* = 0.07).

**Conclusions:**

CVac, a mucin 1-dendritic cell maintenance treatment was safe and well tolerated in ovarian cancer patients. A variable but observed CVac-derived, mucin 1-specific T cell response was measured. Notably, CR2 patients showed an improved PFS and lengthened OS. Further studies in CR2 ovarian cancer patients are warranted (NCT01068509).

**Trial registration:**

NCT01068509. Study Initiation Date (first patient screened): 20 July 2010. Study Completion Date (last patient observation): 20 August 2013, the last patient observation for progression-free survival; 29 April 2015, the last patient was documented regarding overall survival.

## Background

Ovarian cancer is typically managed by surgical cytoreduction followed by platinum and taxane-based chemotherapy. While the majority of patients achieve a clinical remission from this initial therapy, more than 70 % will subsequently develop recurrent disease [1]. Immunotherapeutic approaches to cancer rely on stimulation of the immune system to specifically target and destroy tumors. Mucin proteins are promising targets for immunotherapy; in particular, the epithelial mucin surface antigen 1 is overexpressed in adenocarcinomas in an aberrant form [2–5]. Prior literature have described high levels of MUC1 expression (100 %) in ovarian adenocarcinomas [6] and in late-stage epithelial ovarian cancer (EOC), tumor cells will significantly overexpress mucin 1 showing a significant association between mucin 1 over-expression, histological grade and clinical stage [7, 8]. Therefore, mucin 1 is a specific and appropriate target as an immunotherapy for EOC, whereby the immune system is stimulated to target and destroy tumor cells.

A dendritic cell vaccine targeting the MUC-1 glycoprotein was developed (termed CVac). Prior to the current trial, two clinical trials were conducted with CVac. In the phase I trial, 17 subjects (three healthy volunteers and 14 advanced solid tumor patients) were enrolled, the purpose of which was to establish safety and to observe host immunologic responses. Two of these patients had ovarian cancer, with one patient surviving beyond 12 months post-therapy despite advanced disease. Regarding safety, no anaphylactic reactions were observed and all patients measured a CVac-specific T cell response as measured by ELISpot. No dose limiting toxicities (DLTs) or serious adverse events (SAEs) attributable to CVac were reported in the Phase I study [9].

Subsequently, a phase 2a trial administered CVac to advanced EOC patients who were experiencing progression of disease and for whom standard therapy was no longer available [10]. All 28 patients recruited were evaluable for safety and 26 for efficacy. All had undergone surgery and platinum-based chemotherapy, and 57 % of patients received ≥ 3 chemotherapy regimens. There were no Grade 3 or 4 toxicities considered related to CVac. Despite heavy pre-treatment, four patients showed CA-125 response or stabilization; two patients with major responses defined as > 50 % reduction in CA-125, that were sustained for 10 weeks and 42 weeks, respectively [11]. The median duration was 10.3 months (5.3–16.3 months).

Given the promising results from these trials, a clinical trial with ovarian cancer patients with a low tumor burden following chemotherapy were considered for CVac as a maintenance strategy. Subsequently, the trial CAN-003 was designed as a randomized, multi-center international trial with global manufacturing to evaluate the safety and efficacy of CVac in EOC patients in first (CR1) or second clinical remission (CR2) as a maintenance therapy.

## Methods

### Patients

Patients enrolled into the trial were female ≥ 18 years old with histologically confirmed Stage III or IV epithelial ovarian, primary peritoneal, or fallopian tube cancer who had previously underwent surgical cytoreduction, received first- or second-line conventional chemotherapy, and were in complete remission (CR). CR was defined as no clinical or radiologic evidence of disease and CA-125 below the upper limit of normal (ULN), according to the local laboratory.

CA-125 ≤ upper limit of normal (ULN) with a prior history of an elevated CA-125 was required with not more than 12 weeks between enrollment and the last dose of chemotherapy that resulted in a CR.

Exclusion criteria included ovarian germ cell, sarcoma, or mixed Müllerian tumors, or coexisting or other malignancies unless in CR for at least 3 years, but did not include in-situ carcinoma of the cervix or basal cell or squamous cell carcinoma of the skin, assuming they had been adequately treated. Other exclusion criteria included any active uncontrolled infections or any organ system toxicity ≥ Grade 2 by CTCAE criteria.

### Study design

This was a randomized, open-label, multinational, Phase 2b trial evaluating the safety and efficacy of CVac administered as a single agent for the maintenance treatment of EOC. Eligible patients had achieved CR following conventional chemotherapy and were enrolled after signing informed consents. Ethics and Review board details are at the end of the manuscript.

Manufacturing was transferred from Australia to the US, and as a quality measure, an initial cohort of seven patients who met the study eligibility criteria were not randomized but given one CVac treatment of a total of 6 to 8 injections at four anatomical sites and followed for at least 28 days. This was called the non-randomized CVac cohort (NR-CVac) and they were not included in the efficacy outcome analysis. After the manufacturing characteristics were confirmed to be consistent between facilities, each NR-CVac patient completed one treatment. After 30 days, no serious or treatment-related Grade 3 or 4 adverse events (TEAEs) were reported, patients (*N* = 56) were then enrolled to randomization between standard of care observation versus CVac. All patients who were assigned to receive CVac underwent leukapheresis to collect mononuclear cells (MNCs). Approximately 500 to 800 × 10^6 blood monocytes were collected, and if insufficient cells were obtained, additional leukapheresis procedures were performed. The leukapheresis product was shipped to the manufacturer, and each patient’s MNCs were enriched using cell separation techniques.

For CVac manufacturing, the drug substance, DC-M-FP, was composed of three starting materials: the patient’s autologous dendritic cells (DCs), Mannan (M) and Fusion Protein (FP) to create DC-M-FP. The structural features of each component were: DCs were differentiated from a patient’s peripheral blood mononuclear cells (PBMC or MNC), also referred to as the Mononuclear Cell Product (MNC Product). The MNCs were collected by leukapheresis in certified, inspected and approved leukapheresis units and manufactured under Good Manufacturing Practices (GMP) conditions associated with cells being fractionated and cultured in the presence of cytokines (GM-CSF and IL-4) to promote the differentiation of MNC to DCs. M-FP was the antigen obtained when mannan was oxidized, to form aldehyde groups, and coupled to the FP. M-FP was composed of two parts; (1) mannan, a polymannose with molecular weight of approximately 60 kDa and (2) FP, a recombinant human fusion protein (mucin 1-glutathione S-transferase) with a molecular weight of approximately 38 kDa which was composed of two distinct moieties of a 110 amino acid fragment (12 kDa) of the mucin 1 protein, which was derived from the variable number of tandem repeats (VNTR) region of the mucin 1 protein; and a glutathione S-transferase, a 26 kDa protein.

The patient’s cells were cultured for 5 days with granulocyte-macrophage colony-stimulating factor and interleukin-4 to cultivate the maturation of MNCs to DCs. The culture was treated overnight with the antigen, M-FP, to arm the DCs to the specific mucin 1 antigen creating the DC-M-FP. After washing and formulating in HSA and DMSO at an approximate concentration of 60 × 10^6 DCs/mL, the vials then contained CVac. CVac was cryopreserved and stored in the vapor phase of liquid nitrogen at the manufacturing facility until required [9, 10].

Patients were given 10 doses of CVac, with the first 7 doses administered every 4 weeks and then the last 3 doses administered every 8 weeks. Patients in the SOC group did not undergo leukapheresis nor interventional treatment, but attended the same visit schedule as treated patients.

Patients who completed the first 56 weeks of the study (10 doses of CVac) without disease progression continued to be followed every 12 weeks for an additional 48 weeks until an end-of-study (EOS) PFS visit. All patients were followed for survival until the study closed.

A total of 60 patients were originally planned for recruitment for this study. This sample size for this exploratory Phase 2 study was considered adequate on clinical grounds to evaluate trends in immunological outcomes, disease markers, rates of tumor progression, and other clinical outcomes for the purpose of planning future trials.

### Endpoints and assessments

The primary measure of efficacy was the duration of PFS defined as the date of randomization to the date of documented progression of disease (PD), or death from any cause. PD was defined as either of the following:

- Two serum values of CA-125 ≥ 2× ULN performed at least 1 week apart based on Gynecologic Cancer Intergroup (GCIG) criteria [11]
- Increasing clinical and/or radiological evidence of disease since study entry regardless of serum CA-125 per Response Evaluation Criteria in Solid Tumors (RECIST 1.1) criteria [12]

Secondary endpoints included OS as measured from date of randomization to date of death, from any cause and the evaluation of host immunologic outcomes subsequent to CVac administration.

### Radiological scans

A CT or MRI scan of the chest, abdomen, and pelvis were performed at screening/baseline, after the timing period of three treatments, and every 12 weeks until the end of the study. RECIST version 1.1 guidelines were used to evaluate CT or MRI results for assessment of patient response [11, 12].

### Immunological tests

Serum was collected for assessment of mucin 1 antibodies by ELISA and whole blood for intracellular cytokine staining (ICS) assays at screening/baseline, week 20, and every 12 weeks until the end of the study in both groups [13]. Testing was performed at a central USA laboratory (HIMC, Stanford, CA). For the ICS assays, using eight-color flow cytometry IFNg, TNFa, IL-2, IL-4, and IL-17-producing CD4+ and CD8+ T cells were measured with and without mucin 1 peptide challenge [13, 14].

Mucin 1 antibody and ICS assays were performed to assess the humoral and cellular immunological responses, respectively.

### Safety assessments

Safety was assessed from documentation of AEs, clinical laboratory results (routine hematology, biochemistry and urinalysis), auto-antibody screening, physical examination, vital signs, ECOG status, ECG, and concomitant medications.

## Results and discussion

### Demographics

**Fig. 1:**
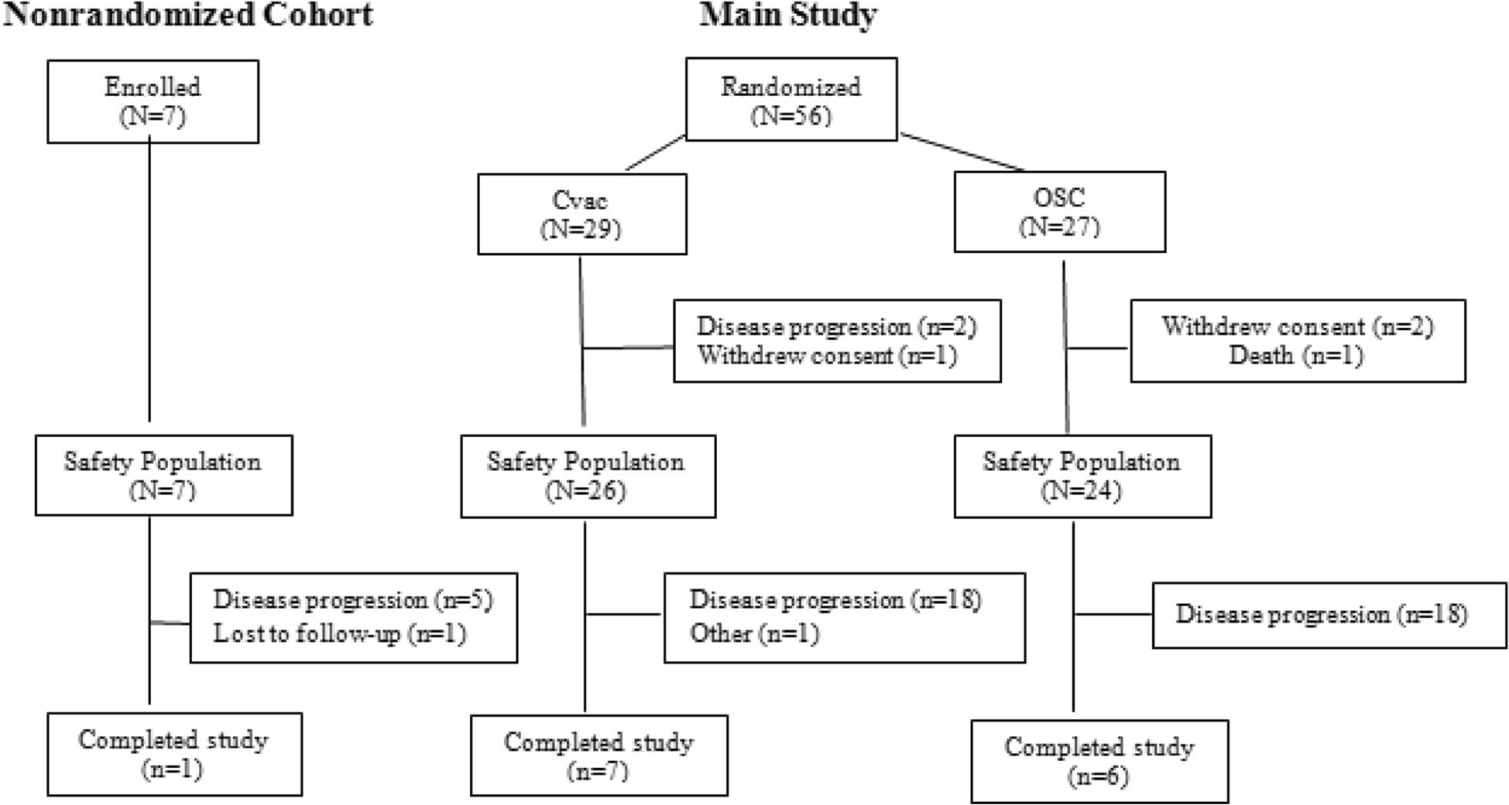
Patient Consort. Patients received 10 doses up till week 56 and were subsequently followed until week 104. Week 104 defined the end of study

Excluding the seven patients treated in the non-randomized lead-in cohort (NR), there were 56 patients randomized to CVac (*N* = 29) and SOC (*N* = 27). Figure 1 outlines patient disposition. Three patients withdrew consent, one patient was lost to follow-up, and one patient was withdrawn for other reasons; none of these five patients had a PFS event. Patients who withdrew consent included 2 SOC patients and 1 CVac patient who did not receive any study treatment. Patient demographics were similar between the patient groups (Table 1).

**Table 1:** Patient Characteristics

	NR	CVac	SOC
	(*N* = 7)	(*N* = 29)	(*N* = 27)
Age, years			
Mean (SD)	52.4 (10.3)	56.8 (8.5)	56.2 (9.5)
Median (min, max)	49 (43–70)	58 (34–75)	55 (40-74)
Race, n (%)			
White	7 (100.0 %)	26 (89.7 %)	23 (85.2 %)
Asian	0	3 (10.3 %)	2 (7.4 %)
Black or African American	0	0	2 (7.4 %)
Ethnicity, n (%)			
Hispanic or Latino	0	0	1 (3.8 %)
Not Hispanic or Latino	7 (100.0 %)	29 (100.0 %)	25 (96.2 %)
Cancer stage at enrollment, n (%)			
III	5 (71 %)	24 (83 %)	20 (74 %)
IV	2 (29 %)	5 (17 %)	7 (26 %)
Histology, n (%)			
Serous	5 (71 %)	25 (86 %)	23 (85 %)
Clear cell	1 (14 %)	0	0
Endometrioid	0	1 (3 %)	2 (7 %)
Mixed, serous	0	1 (3 %)	0
Mucinous	0	1 (3 %)	1 (4 %)
Other	1 (14 %)^a^	0	0
Other, mixed	0	1 (3 %)^b^	1 (4 %)^c^
Cytoreductive Surgery			
Optimal	7 (100 %)	26 (90 %)	21 (78 %)
Sub-optimal	0 (0 %)	3 (10 %)	6 (22 %)

^a^Adenocarcinoma, Primary Mullerian carcinoma

^b^Left ovary high grade adenocarcinoma with mixed clear cell and serous features

^c^Ovarian Adenocarcinoma, Mixed Serous & Transitional cell type Grade 3: solid & focally papillary

Most patients had Stage III cancer at the time of diagnosis and had serous tumor histology (86.2 % in the CVac group and 85.2 % in the SOC group). Cytoreductive surgery was optimal in most patients in the trial with 81 % CR1 and 90 % CR2 patients. All patients received taxane and platinum therapy as first line, with only three patients documented treated with intraperitoneal (IP) chemotherapy.

### Dosing

Of the seven patients in the lead-in, non-randomized cohort (NR), four patients completed the series of ten treatments with CVac. All three patients who did not complete the planned dosing series were discontinued for disease progression, including one patient each who received either 2, 4 or 6 doses.

Of the 26 patients who were randomized to CVac 46 % received all 10 doses while 77 % received 6 or more doses. The remaining breakdown of CVac doses was one patient received 9 doses, five patients received 7 doses, two patients received 6 doses, three patients received 4 doses, two patients received 2 doses, and one patient received 1 dose.

### Safety

Both the randomized and non-randomized cohorts were assessed for safety. Common treatment-emergent AEs (TEAE) seen in greater than 10 % of the patients were unremarkable, similar among groups and generally grade 1–2. Grade 3 or 4 TEAE included:

- EOC metastases to the liver that resulted in discontinuation;
- urinary tract infection, generalized pruritus, cough, headache, and bunion;
- sinusitis, small intestinal obstruction, and disease progression;
- febrile neutropenia;
- small intestinal obstruction;
- abdominal pain, diarrhea, and meniscus lesion (Table 2).

**Table 2:** Treatment-Emergent Adverse Events with Severity Grade 3 or 4 for Patients with CR Following First- or Second-Line Therapy (Safety Population)

	NR-CVac	CVac	SOC	Total
	(*N* = 7)	(*N* = 26)	(*N* = 24)	(*N* = 57)
Patients with TEAE of Severity 3 or 4	0 (0 %)	7 (27 %)	2 (8 %)	9 (16 %)
Small intestinal obstruction	0 (0 %)	2 (8 %)	0 (0 %)	2 (4 %)
Abdominal pain	0 (0 %)	1 (4 %)	0 (0 %)	1 (2 %)
Alanine aminotransferase increased	0 (0 %)	0 (0 %)	1 (4 %)	1 (2 %)
Arthralgia	0 (0 %)	0 (0 %)	1 (4 %)	1 (2 %)
Bunion	0 (0 %)	1 (4 %)	0 (0 %)	1 (2 %)
Cough	0 (0 %)	1 (4 %)	0 (0 %)	1 (2 %)
Diarrhea	0 (0 %)	1 (4 %)	0 (0 %)	1 (2 %)
Disease Progression	0 (0 %)	1 (4 %)	0 (0 %)	1 (2 %)
Escherichia urinary tract infection	0 (0 %)	1 (4 %)	0 (0 %)	1 (2 %)
Febrile neutropenia	0 (0 %)	1 (4 %)	0 (0 %)	1 (2 %)
Gamma-glutamyltransferase increased	0 (0 %)	0 (0 %)	1 (4 %)	1 (2 %)
Hand fracture	0 (0 %)	0 (0 %)	1 (4 %)	1 (2 %)
Headache	0 (0 %)	1 (4 %)	0 (0 %)	1 (2 %)
Meniscus lesion	0 (0 %)	1 (4 %)	0 (0 %)	1 (2 %)
Metastases to liver	0 (0 %)	1 (4 %)	0 (0 %)	1 (2 %)
Ovarian epithelial cancer metastatic	0 (0 %)	1 (4 %)	0 (0 %)	1 (2 %)
Pruritus generalized	0 (0 %)	1 (4 %)	0 (0 %)	1 (2 %)
Sinusitis	0 (0 %)	1 (4 %)	0 (0 %)	1 (2 %)

The investigator associated causality of these TEAEs stated that generalized pruritus and headache were considered probably and possibly related to CVac, respectively. Seven patients had a total of 9 SAEs during the study (4 CVac patients 3 SOC patients), including one SAE in a SOC patient resulting in death (subdural hematoma in a patient with a medical history of hypertension, right ventricular thrombus and myocardial infarction). The 4 CVac patients with SAEs included abdominal pain and metastatic EOC; small intestinal obstruction and disease progression; disease progression with later hospitalization for febrile neutropenia was due to subsequent chemotherapy after progression on CVac. Progression of metastatic EOC in the patient resulted in interruption of treatment and discontinuation from the study. All SAEs were considered unrelated to the treatment.

One patient was discontinued from the study for AE of EOC metastases to the liver that occurred 13 months after the first dose and 2 months after the last dose of CVac treatment. The AE of EOC metastases was considered unrelated to CVac.

### Immuno-assay

Mucin 1 antibodies measured from blood samples throughout the study were assessed by a quantitative ELISA [13]. Mean mucin 1 antibody levels were low and did not notably change with treatment throughout the study. Only two patients who were in the SOC group showed a low anti-mucin 1 response possibly as a result of an endogenous response to tumor expression. No anti-mucin 1 immune activity was measured in the CVac treated patients (data not shown).

**Fig. 2:**
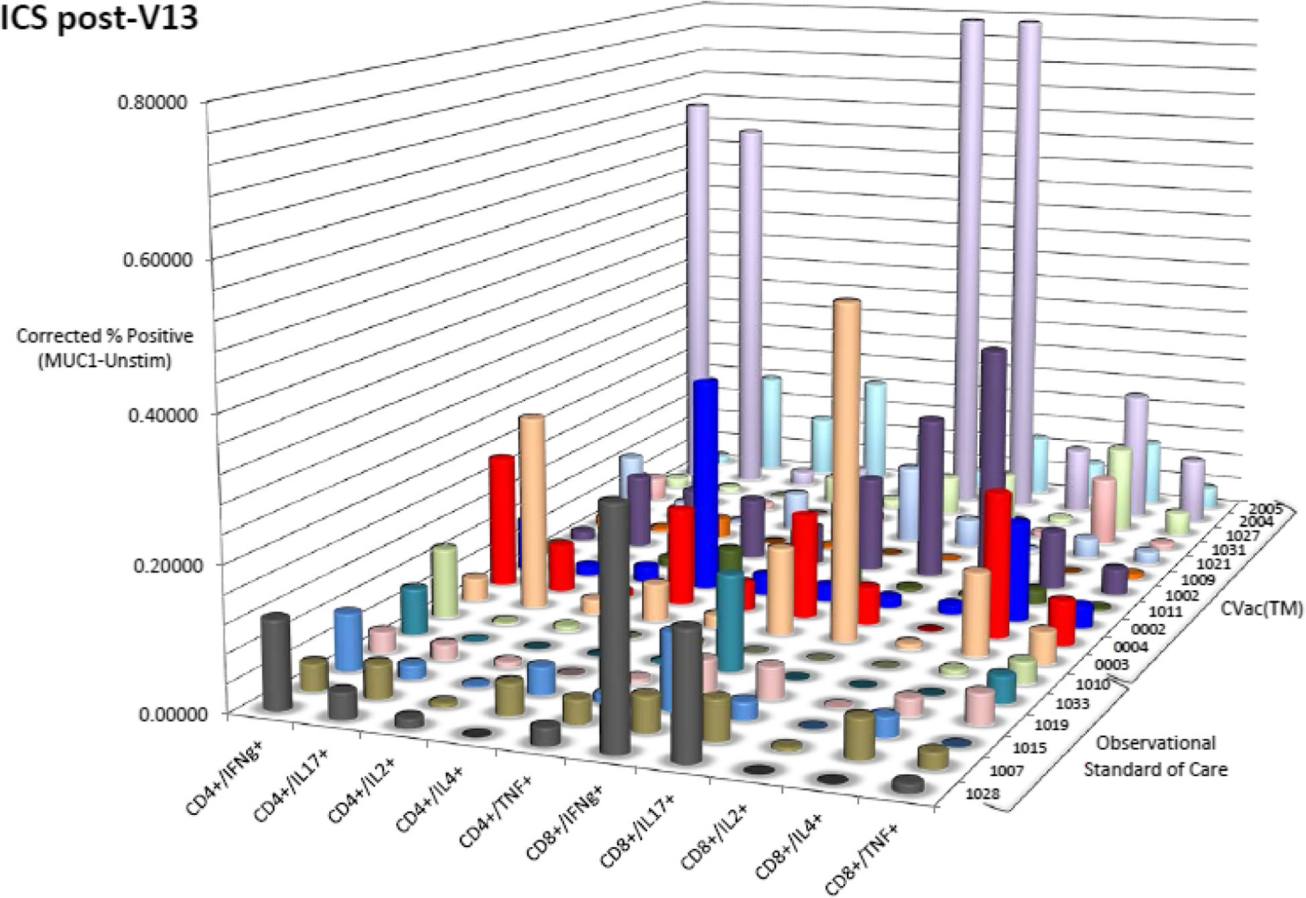
Intracellular cytokine staining (ICS) of CVac-treated patients. Values are corrected for the background (endogenous unstimulated response) subtracted from the mucin 1 (MUC1) challenge shown as a percentage. Values of zero or less are shown as zero. The samples shown represent those patients that had both a baseline sample and a visit 13 sample (completion of 10 doses of CVac treatment) available for analysis

To assess T cell responses, intracellular cytokine staining data were measured from PBMC at screening/baseline, Week 20, and every 12 weeks until the end of the study in both groups. SOC patients’ T cells measured a small or absence of response when challenged with mucin 1 [14]. However, CVac-treated patients had T cells that responded to mucin 1 challenge seen with both CD4+ (helper T cells) and CD8+ (killer T cells). In this cohort of patients, CD8+ cytotoxic T cells showed a greater reactivity than CD4+ T helper cells. Kinetics of intracellular cytokine (ICS) expression were difficult to determine due to a wide variability in this small patient cohort. At the end of dosing (visit 13 after 10 doses in total; Fig. 2) there remained a detectable mucin 1-specific T cell response in treated patients as compared to SOC that was measurable over endogenous baseline (unstimulated), which suggested a prolonged immune response.

### Efficacy

#### Progression free survival

At the end of 48 weeks of PFS follow up, CVac treated patients showed a median PFS of 13 months as compared to 9 months for the SOC group (hazard ratio [HR] of 0.72, *p* = 0.33 CI 0.38–1.38). Given the heterogeneity in the enrolled population, subgroup analysis was performed to determine if there was a differential survival benefit for CVac treated patients based on their remission status (CR1 vs. CR2).

**Fig. 3:**
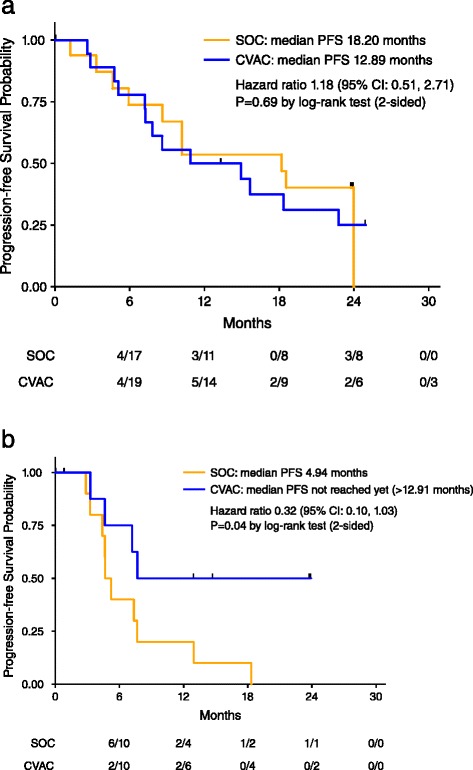
Progression-free survival. Progression-free survival was defined as the time from date of randomization to the earlier of disease progression or death due to any cause. Vertical tick marks represent the progression-free survival time of patients without disease progression. Hazard ratio (CVac/SOC) was estimated using a Cox proportional hazards model. *P*-value was calculated using the log-rank test. **a** Progression-free survival for patients in complete remission after first-line therapy; CR1. **b** Progression-free survival for patients in complete remission after second-line therapy; CR2

Analysis of the patients in CR1 revealed no significant difference in PFS between the CVac and SOC subjects (13 vs. 18 months) (HR = 1.18; CI 0.52–2.71; *p* = 0.69) (Fig. 3). However, as patients were enrolled within 12 weeks of their last chemotherapy, evaluation of the PFS curves suggested that 8 of 36 (22 %) patients progressed at ≤ 6 months, consistent with primary platinum-resistance in the CR1 cohort. In the CR2 cohort the median PFS for CR2 patients treated with CVac (*N* = 10) was greater than 13 months; compared to the SOC control group (*N* = 10) was 5 months, an observed hazard ratio of 0.32 (CI 0.10–1.03; *p* = 0.04).

#### Overall survival

**Fig. 4:**
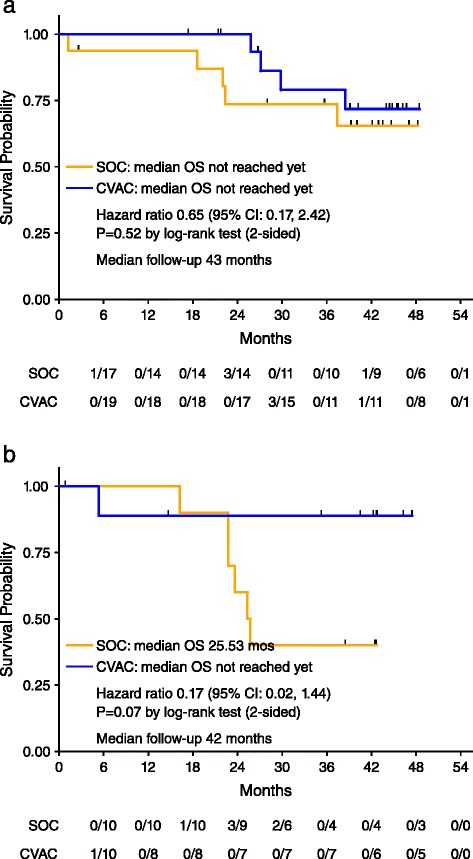
Overall Survival. Overall survival was defined as the time from date of randomization to the date of death due to any cause. Vertical tick marks represent the overall survival time of patients reported alive or lost to follow-up as of the last contact. Hazard ratio (CVAC/OSC) was estimated using a Cox proportional hazards model. *P*-value was calculated using the log-rank test. **a** Overall survival for patients in complete remission after first-line therapy; CR1. **b** Overall survival for patients in complete remission after second-line therapy; CR2

OS was followed until 43 months and the median OS was not reached in either arm for the overall population (HR = 0.38, *p* = 0.06, 95 %; data not shown). Sub-analysis of remission status showed in CR1 patients that after 40.5 months of follow up, a median OS was not reached [15] (Fig. 4). CR2 patients at 42 months of follow up showed that the SOC patients had attained a median OS of 26 months consistent with general literature estimates of 24–29 months [15–17]. Patients administered CVac had not reached median overall survival, which suggested an increase of > 16 months in this cohort.

## Conclusion

The treatment of EOC has been filled with approaches that have resulted largely in PFS benefit in the biologic era with front-line maintenance +/−concurrent anti-VEGF therapies (bevacizumab, pazopanib, nintedanib [15–18]) but not median overall survival (OS) benefit to date. Some will argue that the availability of multiple lines of subsequent therapies and cross-over effect may account for this observation. The only randomized phase 3 trial to show an preliminary OS benefit to date with a multi-targeted anti-VEGF agent (cediranib) is ICON-6, in the recurrent platinum-sensitive setting. There are also phase 3 trials in the concurrent and/or switch-maintenance setting with poly-ADP ribose polymerase inhibitors (PARPi) in the high-grade serous and endometriod histological sub-groups; these trials are ongoing or awaiting maturity [19–22]. As we move into the era of immuno-oncology we are hopeful that a clearly demonstrable OS benefit is finally within grasp in the near future.

This trial evaluated CVac, a DC immunotherapeutic approach targeting mucin 1 in EOC. EOC patients in first and second clinical remission had immune systems that elicited a cellular, mucin 1 specific T cell response and not a humoral response to treatment. CVac was well tolerated and safe with the majority of TEAE as grade 1–2 with similar distribution in both groups (fatigue, abdominal pain, diarrhea and nausea).

Although this study was designed as an exploratory phase 2 trial, overall we found patients treated with CVac compared to observation had no improvement in PFS or OS. However, in the sub-group analysis, patients in CR2 treated with the dendritic cell vaccine, CVac, showed an improvement in both PFS and OS. Additionally, we observed the PFS curves in CR2 patients to have a typical shape in cancer immunotherapy. First, there was the classic delayed effect with no difference between treatment arms during the first 4–5 months of the study. Then, we observed a significant “long tail” indicating that the CVac patients who may have responded stayed in remission for an extended time. Notably, this study was small, with inherent biases of a randomized phase 2 trial; therefore a larger phase 3 trial is required to be more definitive.

To explain the survival differences observed between CR1 and CR2 patients with maintenance CVac, we hypothesized that the CR2 group of patients was a much more homogenous group, and all likely primary platinum sensitive. We noted a 22 % primary platinum resistance in the CR1 patients. CR2 patients may have represented a better prognostic group overall and perhaps responded more likely to immunotherapy as a maintenance strategy. Neither Provenge nor Yervoy (two approved immunotherapeutics for metastatic prostate cancer and melanoma, respectively) demonstrated a difference in PFS as compared to the control arm in their phase 3 trials; both demonstrated clinically significant and statistically significant differences in OS.

Several recently presented phase 3 trials with immune check-point inhibitors (largely with anti-PD1 antibodies, nivolumab or pembrolizumab); have shown a significant prolongation in OS in metastatic melanoma, clear-cell renal cell carcinoma (2^nd^-line) and non-small cell lung cancer, further supporting the role of immunotherapeutics in solid tumors. Immunotherapies have become an important part of treating several types of cancer; the indication for which is growing. The safety profile shown with CVac, and the improved outcomes of PFS and OS were encouraging and worthy of further study.

## Abbreviation

AE: adverse event
CR1: first clinical remission
CR2: second clinical remission
CVac: DCs treated with a mannosylated recombinant human fusion protein comprised of mucin 1- glutathione S-transferase
DC: dendritic cell
EOC: epithelial ovarian cancer
HR: hazard ratio
ICS: intracellular cytokine staining
IFNγ: interferon gamma
IL: interleukin
M-FP: mannosylated recombinant human fusion protein comprised of mucin 1-glutathione S-transferase
NR: non randomized
OS: overall survival
PBMC: peripheral blood mononuclear cel
PD: progressive disease
PFS: progression-free survival
SAE: serious adverse event
SOC: standard of care
TEAE: treatment emergent adverse event
TNFα: tumor necrosis factor alpha
ULN: upper limit of normal
